# Short-Term Variations of C18:1 *Trans* Fatty Acids in Plasma Lipoproteins and Ruminal Fermentation Parameters of Non-Lactating Cows Subjected to Ruminal Pulses of Oils

**DOI:** 10.3390/ani11030788

**Published:** 2021-03-12

**Authors:** Einar Vargas-Bello-Pérez, Juan J. Loor, Philip C. Garnsworthy

**Affiliations:** 1 Department of Veterinary and Animal Sciences, Faculty of Health and Medical Sciences, University of Copenhagen, Grønnegårdsvej 3, DK-1870 Frederiksberg C, Denmark; 2 School of Biosciences, Sutton Bonington Campus, University of Nottingham, Loughborough LE12 5RD, UK; Phil.Garnsworthy@nottingham.ac.uk; 3 Department of Animal Sciences and Division of Nutritional Sciences, University of Illinois, 1207 West Gregory Drive, Urbana, IL 61801, USA; jloor@illinois.edu

**Keywords:** biohydrogenation, cows, lipoproteins, ruminal fluid, *trans* fatty acids, vaccenic acid

## Abstract

**Simple Summary:**

The objective of this study was to evaluate short-term variations of *trans* fatty acids (TFA) in plasma lipoproteins and ruminal fermentation parameters of non-lactating cows subjected to ruminal pulses of vegetable oils. Over three-day periods, three non-lactating, non-pregnant Holstein cows received ruminal pulses of soybean oil, partially hydrogenated vegetable oil and a control with no added oil. In summary, results showed that for both oil treatments there was an accumulation of several C18:1 TFA in plasma and lipoproteins, especially on the third day of pulsing. Each C18:1 TFA responded differently to treatments over time.

**Abstract:**

The objective of this study was to evaluate short-term variations of *trans* fatty acids (TFA) in plasma lipoproteins and ruminal fermentation parameters of non-lactating cows subjected to ruminal pulses of vegetable oils. Three non-lactating, non-pregnant Holstein cows, each with a ruminal cannula, were arranged in a 3 × 3 Latin square design with three-day pulsing periods and four-day washout intervals between treatments. Cows were treated with single ruminal pulses of: (1) control (skimmed milk (SM); 500 mL); (2) soybean oil (SO; 250 g/d in 500 mL of SM) and (3) partially-hydrogenated vegetable oil (PHVO; 250 g/d in 500 mL of SM). Time changes after infusion in TFA contents were only observed for plasma C18:1 *trans*-4, *trans*-5 and *trans*-12, and high-density lipoprotein fraction C18:1 *trans*-9. After ruminal pulses, concentration of acetate decreased linearly; molar concentrations of propionate and valerate increased linearly; molar concentrations of butyrate and isovalerate changed quadratically and were greater at 1 h than at other times. There was an accumulation of several C18:1 TFA in plasma and lipoproteins, especially on the third day of pulsing. Overall, naturally occurring C18:1 TFA isomers (produced during ruminal biohydrogenation of SO) and preformed TFA (supplied by PHVO) elicited differential TFA partitioning and transport in plasma and lipoproteins.

## 1. Introduction

*Trans* fatty acids (TFA) are produced naturally during biohydrogenation of polyunsaturated fatty acids (FA) in the rumen and are incorporated into milk fat [1]. The predominant TFA in ruminant fat is vaccenic acid (C18:1 *trans*-11), accounting for 60% to 80% of total TFA, although other TFA (e.g., C18:1 *trans*-10) occur when high-fat or high-concentrate diets are fed to ruminants. Partially hydrogenated vegetable oils (PHVO) contain a mixture of C18:1 *trans* isomers [2] and their proportions vary depending on the source of vegetable oil. In fact, PHVO from industrial origin contain up to 50% TFA, mainly C18:1 *trans*-9 and C18:1 *trans*-10 [3]. Some TFA, such as C18:1 *trans*-11, have been reported to be beneficial for human health [4] and their concentrations can be increased in milk by high grass intakes [5], or by supplementing dairy cow diets with vegetable oils such as soybean [6], linseed, rapeseed and sunflower oils [7].

Cholesterol is important for plasma transport of FA in all mammals and plays vital roles in cell membrane structure and steroid hormone synthesis [8]. Although proportions of low- and high-density lipoproteins (LDL and HDL) have been studied extensively in humans, lipoproteins have received little attention in ruminants. The mammary gland appears to have differential uptake of lipids from lipoprotein fractions, which affects efficiency of transfer of long-chain fatty acids (LCFA; >18 carbon atoms) from diet to milk [9]. Sun and Gibbs [10] reported that FA profiles in ruminal digesta vary during the day, reflecting microbial colonization of newly ingested feed affecting the biohydrogenation pathways in grazing cattle. Loor et al. [11] suggested that ruminal pH changes and milk FA may be related, and Colman et al. [12] reported this with a logistic curve showing the link between those parameters. There are studies [13,14] reporting the effect of different dietary oils on FA transport in lipoprotein fractions of lactating and non-lactating cows. Those studies reported differences in the transport of FA in plasma lipoproteins over 21-day periods but did not evaluate short-term variations in C18:1 TFA in plasma lipoproteins. Evaluating short-term variations explains immediate responses to FA supply that provoke energy status changes, which are closely related to ruminal fermentation byproducts, such as production of propionic acid [15]. Also, dietary oils can result in extensive biohydrogenation effects that are often observed during the first few hours after administration [16].

From a productive perspective, feeding different sources of vegetable oils may be used to increase some TFA, such as C18:1 *trans*-11 in milk [6,17,18], that are beneficial for human health, so it is important to understand time variations in C18:1 TFA that might arise from this approach. During the periparturient period, when cows are particularly susceptible to infection and inflammation, shifts in the composition of plasma lipids are coupled with changes in the FA composition of immune cells [19]. Compared with naturally occurring *cis* unsaturated FA, *trans* double bonds result in FA structurally analogous to saturated fats, leading to reduced cell membrane fluidity and possibly altered cell signaling. When included in the diet, therefore, TFA have the potential to integrate into cell membranes and alter immune cell function through various processes, including alteration in expression of genes encoding for pro-inflammatory mediators [20].

From a metabolic perspective, feeding non-lactating cows with different FA sources can be a strategy for priming lipid metabolism in dairy cows to cope with the metabolic challenges in the following early lactation phase [21]. In this regard, previous studies have fed dry cows with different FA sources focused on postpartum productive [21,22,23] and reproductive traits [24,25]; however, no report is available on dry cows’ short-term metabolism of TFA.

Identifying times when lipid supplements are less affected by ruminal biohydrogenation or have less effect on ruminal fermentation parameters (e.g., pH, NH_3_-N and volatile fatty acids) is valuable information for ruminant nutritionists. In this regard, studies in which oils are ruminally infused as pulses are limited. Due to the aforementioned background, before performing large, controlled feeding trials and on-farm experiments in lactating animals, the approach of this study was to analyze the short-term effects of different FA sources on blood lipoproteins and rumen fermentation parameters in non-lactating dairy cows. Another feature of our approach was to consider how C18:1 TFA are partitioned and transported into lipoproteins. Therefore, the objective of this study was to evaluate the short-term variations of C18:1 TFA in plasma lipoproteins and ruminal fermentation parameters of cows subjected to ruminal pulses of vegetable oils.

## 2. Materials and Methods

### 2.1. Animals and Treatments

All procedures were reviewed by the Animal Welfare and Ethical Review Board of the University of Nottingham and were conducted in accordance with the requirements of the UK Home Office Animals (Scientific Procedures) Act 1986 under project licence ID 40/2751. Three non-lactating, non-pregnant Holstein multiparous cows (BW = 773 ± 63 kg (average ± SD)), each with a ruminal cannula, were utilized in a 3 × 3 Latin square design with a three-day treatment period followed by a four-day washout interval between treatments to minimize carryover effects. The periods were short because the effect of FA and energy absorption were assumed to regulate ruminal fermentation parameters in the short-term [15].

The basal diet consisted of meadow hay (*ad libitum*; approximately 8 kg/d) and a commercial concentrate (2 kg/day). Meadow hay contained 87% dry matter (DM), 9.3% DM crude protein, 63% DM neutral detergent fiber, 38% DM acid detergent fiber and 1.6% DM ether extract, as determined in a commercial feed laboratory. The basal diet was formulated to meet 110% of nutrient requirements for maintenance of a non-lactating, non-pregnant cow weighing 750 kg [26]. Ingredients and chemical composition of the commercial concentrate are shown in Table 1. The animals had ad libitum access to hay, and the commercial concentrate was offered at 12:00 (3 h after the pulse of lipids). Cows were dosed at 09:00 with ruminal pulses of: (1) control (skim milk (SM); 500 mL/d); (2) soybean oil (SO; 250 g/d in 500 mL/d of SM) and (3) partially hydrogenated vegetable oil (PHVO; 250 g/d in 500 mL/d of SM). PHVO was supplied by Aarhus Karlshamn Ltd. (Hull, UK) and was based on hydrogenated rapeseed oil. Quantities of fat and SM in ruminal pulses were calculated according to previous studies [27]. The control supplied 3% (of total diet DM) of ether extract while SO and PHVO supplied 4% (of total diet DM) of ether extract.

During pulses and sampling periods, cows were housed in tie-stalls and had free access to water. Treatment oil emulsions were delivered through the rumen cannula as ruminal pulses once daily at 09:00 over three days. Emulsions were prepared the day before each pulse. To provide a homogenized release of pulses, skim milk was used (500 mL/d) to emulsify the oils (SO and PHVO) using a laboratory mixer emulsifier (L2R Silverson, Machines Ltd., Waterside, Chesham, UK). Before each pulse, emulsions were heated for 30 min at 30 °C in a water bath (Grant Y28, Cambridge Ltd., Cambridge, UK) to provide easier diffusion of PHVO. The FA profile of treatment oils and emulsions is shown in Table 2.

### 2.2. Ruminal Fluid Samples

Samples of ruminal fluid (50 mL) were collected from various parts (around 17 mL per site) of the rumen (anterior, dorsal and mid-ventral regions) using a syringe screwed to a stainless tube. Ruminal fluid samples were collected prior to ruminal pulse (0 h; 09:00) and at 1 (10:00), 3 h (12:00) and 6 h (15:00) post pulsing during the 3 d collection periods. Immediately after fluid collection, a 10 mL aliquot of ruminal fluid was used to determine pH by using a pH meter (Piccolo Plus, HI 1295, Hanna Instruments, Póvoa de Varzim, Portugal). Following pH determination (recorded at 0, 1, 2, 3 and 6 h), ruminal fluid was centrifuged at 20 °C for 10 min at 3000× *g* (Centaur 2 MSE, Milton Keynes, UK). Immediately after rumen fluid collection, a 10 mL aliquot of ruminal fluid supernatant was preserved by adding 1 mL of 25% metaphosphoric acid for volatile fatty acid (VFA) determination, and another 10 mL aliquot of untreated ruminal fluid was kept for NH_3_-N analysis. Analyses of VFA and NH_3_-N were only done at 0, 1, 2 and 3 h. Samples were immediately frozen at −20 °C for later analysis.

Samples of ruminal fluid were analyzed for VFA using a gas chromatograph (GC; Agilent, GC 6890 series, Stockport, UK) equipped with a CP-Sil 88 fused-silica capillary column (100 m × 0.25 mm i.d., with 0.2-µm film thickness; Varian Inc., Oxford, UK). A flame ionization detector was used with an oven initial temperature of 140 °C and maximum temperature of 250 °C and an equilibrium time of 0.50 min. The inlet temperature was 300 °C, the split ratio was 15:1 and a 1-µL injection volume was used. The detector temperature was 250 °C, hydrogen carrier gas flow to the detector was 40.0 mL/min, airflow was 450 mL/min and the flow of nitrogen makeup gas was 45.0 mL/min. A volatile free acid mixture standard (Supelco-46975-U; Sigma-Aldrich, Gillingham, UK) was used for VFA determination. Ruminal NH_3_-N was determined by a photometric test with a Clinical Chemistry Auto Analyzer using an enzymatic ultraviolet method (Rx Imola; Randox Laboratories Ltd., Cat. No. AM3979, Crumlin, UK).

### 2.3. Plasma Samples

Blood samples (10 mL) were obtained via jugular catheter (placed the day before sampling periods) prior to the pulse (0 h) and at 1, 2, 3 and 6 h after pulsing during each three-day collection period for lipoprotein fractionation. Immediately after sampling, blood was transferred to EDTA tubes and centrifuged at 20 °C for 10 min at 3000× *g* (Centaur 2 MSE, Milton Keynes, UK) for harvesting plasma. Based on their density, lipoprotein fractions were separated sequentially by preparative ultracentrifugation in a Beckman XL-70 preparative centrifuge (Beckman Coulter UK Ltd., High Wycombe, UK). Potassium bromide was added to the plasma to obtain the required density calculated by use of the Radding and Steinberg [28] formula. Plasma was separated into lipoprotein fractions (LDL and HDL fractions) by ultracentrifugation at 39,000× *g* for 20 h at 12 °C. Tubes were carefully removed after ultracentrifugation and each tube was cut using a Kontron bench tube slicer to retrieve the top layer, which was predominantly a LDL fraction. The density of lipoprotein and KBr solution was re-adjusted to obtain the HDL fraction and tubes were ultracentrifuged at 39,000× *g* for 40 h at 12 °C. Tubes were carefully removed from the centrifuge and cut as previously described.

### 2.4. Fatty Acid Analysis

Lipids from plasma and lipoprotein fractions were extracted according to Hara and Radin [29] by using hexane:isopropanol (3:2) and sodium sulfate; then transmethylated with 0.5 M sodium methoxide/methanol following chemical derivatization with 50% BF3-MeOH. All chemicals and solvents used for this method were of analytical grade. For analysis of FA in plasma and lipoprotein fractions, a GC system (Agilent, GC 6890 series, Stockport, UK) equipped with a CP-Sil 88 fused-silica capillary column (100 m × 0.25 mm i.d., with 0.2 μm film thickness; Varian Inc., Oxford, UK) was used. The GC conditions were as follows: the oven temperature was initially set at 110 °C for 4 min after injection and then increased to 240 °C with equilibration time of 2 min. The inlet and flame-ionization detector temperatures were 260 °C, the split ratio was 15:1 and a 2-μL injection volume was used. The hydrogen carrier gas flow to the detector was 25 mL/min, airflow was 400 mL/min, and the flow of nitrogen makeup gas was 40 mL/min. Fatty acid peaks were identified by using a fatty acid methyl ester standard (FAME; Supelco 37 Component FAME mix, Bellefonte, PA, USA) and a TFA reference standard (C18:1 *trans*-11, methyl ester, Supelco, Bellefonte, PA, USA).

### 2.5. Statistical Analysis

Animals were arranged in a 3 × 3 Latin square design and data were analyzed as repeated measurements across time using the Genstat statistical package (19th Edition; VSN International Ltd., Oxford, UK) with the following model:

Y_ijkl_ = µ + T_i_ + P_j_ + C_k_ + S_l_ + D_t_ + T_i_ × S_l_ + T_i_ × D_t_ + e_ijkl_

where Y_ijkl_ is the observation, µ is the population mean, T_i_ is the treatment (i = control, SO or PHVO), P_j_ is the period (j = 1, 2 or 3), C_k_ is the random effect of cow (k = 1, 2 or 3), S_l_ is the sampling day within the period (l = 1, 2 or 3), D_t_ is the sampling time within day (1, 2, 3, 4 or 6 h), T_i_ × S_l_ is the treatment × sampling day interaction, T_i_ × D_t_ is the treatment × sampling time interaction and e_ijkl_ is the residual error, which includes all other interactions, none of which were significant. Tukey’s test was used to identify significant differences between means. Probability of *p* < 0.05 was used to declare significant differences among means. Results are reported as least-square means for effects of interest, with observations pooled to ensure appropriate error terms for each comparison. Thus, day means are for pooled times, time means are for pooled days, and *n* = 3 for each individual mean.

## 3. Results and Discussion

### 3.1. Fatty Acid Profile of Oils and Emulsions

The most abundant FA in dietary oils were the following: SO contained (g/100 g of total FA) 22 of C18:1 *cis*-9 and 53 of C18:2 *cis*-9, 12 (no C18:1 TFA were detected in this oil); PHVO contained (g/100 g of total FA) 8 of C16:0 and 8 of C18:0. The PHVO also contained (g/100 g of total FA) some C18:1 TFA: 0.59 of C18:1 *trans*-4, 1.48 of C18:1 *trans*-5, 18 of C18:1 *trans*-6–8, 8 of C18:1 *trans*-9, 9 of C18:1 *trans*-10, 8 of C18:1 *trans*-11 and 7 of C18:1 *trans*-12. The SO emulsion contained a high proportion of polyunsaturated FA, mainly C18:2 *cis*-9, *cis*-12 (~50 g/100 g) and C18:1 *cis*-9 (~20 g/100 g). The PHVO emulsion contained a high proportion (~50 g/100 g) of TFA covering the range of C18:1 *trans* positional isomers from *trans*-4 to *trans*-12. C18:1 *trans* isomers were not detected in the SO emulsion while control only presented C18:1 *trans*-11.

### 3.2. Ruminal Fermentation Parameters

There was no treatment by period interaction in any of the fermentation parameters. Ruminal NH_3_-N concentration (mmol/L) was higher (*p* < 0.05) for control (15) and PHVO (13) than for SO (8). Total VFA concentration (mmol/L) in ruminal fluid was lower (*p* < 0.05) for PHVO (72), and SO (72) than control (90). Ruminal pH and molar percentages of acetate, propionate, butyrate, isobutyrate, valerate and isovalerate were not affected (*p* > 0.05) by treatments. Dietary lipids can reduce fiber digestibility and alter ruminal fermentation parameters [27]. Ruminal pulses of SO and PHVO decreased (*p* < 0.05) total ruminal VFA concentration; this effect may be related to a reduced availability of rapidly fermentable carbohydrate in the rumen [30] or a decrease in fiber digestion [31].

Ruminal pulses of SO decreased (*p* < 0.05) NH_3_-N concentration. It is possible that SO reduced availability of fermentable carbohydrates for microbial synthesis [32]. Availability of fermentable carbohydrates would be reduced if SO physically coated dietary fiber and concentrates, thus making them inaccessible to microbial attack. Less physical coating would be expected with PHVO due to its higher melting point compared with SO. Introduction of one *cis* double bond into a FA chain reduces melting point considerably compared to saturated FA chains with the same number of carbon atoms [33]. In a previous experiment where cows were supplemented with 3% (DM basis) of SO or PHVO, Vargas-Bello-Pérez et al. [34] reported no effect on NH_3_-N and total concentration of VFA.

Effects of dietary oil supplementation on ruminal NH_3_-N concentration can be variable and other factors are related to NH_3_-N production: protozoa counts and contents of proteolytic bacteria [35]. In a meta-analysis, it was reported that rumen protozoa decrease when ruminants are fed with lipid supplements and that this is due to changes in membrane permeability [36]. Conversely, protozoa membranes are more susceptible to the action of medium-chain FA such as palmitic acid [37], which was present in both oils used in this study. Thus, in the present study, concentration of NH_3_-N may have decreased due to exclusion of protozoal proteolytic and deamination enzymes [38].

Ruminal pH and NH_3_-N did not differ (*p* > 0.05) between days. Ruminal pH decreased linearly (*p* < 0.05) from 6.62 to 6.27 and was lower at 6 h post-rumen pulsing than at other times. This agrees with the decrease of ruminal pH (from 6.30 to 5.70) reported by Gustafsson and Palmquist [39] when cows (fed corn silage, alfalfa hay and concentrate) were sampled throughout 16 h post-feeding. Sun and Gibbs [10] reported the lowest rumen pH value (6.07) at night (from 18:00 to 6:00). Under the conditions of our study, the drop in rumen pH (lowest at 6 h post-rumen pulse) could be related to concentrate feeding rather than the lipid pulses.

**Figure 1:**
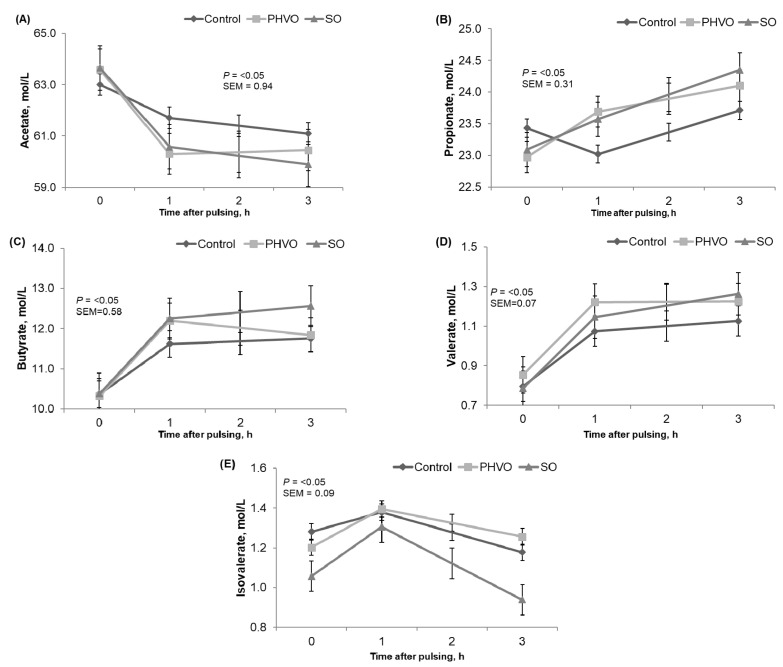
Time variations of volatile fatty acids from cows subjected to ruminal pulses of control, partially hydrogenated vegetable oil (PHVO) and soybean oil (SO). (**A**) Acetate; (**B**) propionate; (**C**) butyrate; (**D**) valerate and (**E**) isovalerate. Ruminal fluid samples were collected prior to ruminal pulse (0 h; 09:00) and at 1 and 3 h after ruminal pulse (10:00 and 12:00). SEM = standard error of the mean. Probability of *p* < 0.05 was used to determine significant differences between means. Means are pooled across days for each cow. *p*-value represents the effect of sampling time.

Ruminal NH_3_-N increased quadratically (*p* < 0.05) from 11 to 17 mmol/L and was greater (*p* < 0.05) at 3 h post-rumen pulsing than at other times. Total VFA were not affected (*p* > 0.05) by day or time of sampling. Concentrations of acetate, propionate, butyrate, valerate and isovalerate did not differ between days. Concentration of acetate decreased linearly (*p* < 0.05); molar concentration of propionate and valerate increased linearly (*p* < 0.05); and molar concentrations of butyrate and isovalerate decreased quadratically (*p* < 0.05) and were greater (*p* < 0.05) at 1 h post-rumen pulsing than at other times, but molar concentration of valerate was not affected by time of sampling (Figure 1). As reported by Gustafsson and Palmquist [39], in the current study concentrations of propionate and butyrate increased, whereas acetate decreased after the first hours post daily morning feeding.

### 3.3. Treatment Effects on Plasma and Lipoprotein Fractions

There was no treatment by period interaction for any FA in plasma and lipoproteins. Compared with control and SO, PHVO increased (*p* < 0.05) plasma concentrations (g/100 g of total FA) of C18:1 *trans*-4 (0.001 and 0.001 vs. 0.2) and C18:1 *trans*-5 (0.1 and 0.1 vs. 0.2). However, C18:1 *trans*-12 was higher (*p* < 0.05) in SO and PHVO than control (0.3 vs. 0.3 and 0.2). As expected, we detected higher concentrations of C18:1 *trans* isomers in PHVO pulses.

Concentrations (mg/L) of HDL and LDL fractions were not affected by treatment (*p* > 0.05) (0.4 ± 0.05 mg/L for HDL and 0.1 ± 0.002 mg/L for LDL). Compared with control, SO and PHVO increased (*p* < 0.05) HDL content of C18:1 *trans*-9 (0.01 and 0.02 vs. 0.09 g/100 g of total FA). This TFA has been reported as one of the most predominant C18:1 *trans* isomers in PHVO [2]. Compared with control and PHVO, SO resulted in higher (*p* < 0.05) concentrations of C18:1 *trans*-11 (0.5 and 0.5 vs. 0.7) in the LDL fraction. Compared with control and SO, PHVO resulted in higher (*p* < 0.05) concentrations (g/100 g of total FAs) of C18:1 *trans*-5 (0.04 and 0.09 vs. 0.2), C18:1 *trans*-9 (0.09 and 0.2 vs. 0.5), and C18:1 *trans*-10 (0.08 and 0.1 vs. 0.5) in the LDL. Compared with control and PHVO, SO resulted in higher (*p* < 0.05) concentrations of C18:1 *trans*-11 (1.1 and 1.3 vs. 2.6) in the LDL fraction. The increased contents of C18:1 *trans*-11 in both HDL and LDL fractions by SO are in agreement with previous studies [13,34]. This is because unsaturated FA sources such as SO lead to formation and accumulation of C18:1 *trans*-11 in the rumen [40].

In general, PHVO increased most C18:1 *trans* isomers in the LDL fraction, some of which have been negatively correlated with milk fat synthesis (i.e., C18:1 *trans*-10 and C18:2 *trans*-10, *cis*-12). In this regard, our gas chromatography protocol was able to detect C18:1 *trans*-10. This TFA deserves special attention since it is related to milk fat depression in lactating ruminants [41] and can affect expression of several genes involved in lipid metabolism in the mammary gland [42]. This study agrees with previous reports where the LDL fraction appears to be the main lipoprotein transporting C18:1 *trans* isomers and is more responsive than other lipoprotein fractions to variation in supply of dietary lipids [14].

### 3.4. Plasma and Lipoproteins: Day and Time Changes

**Figure 2:**
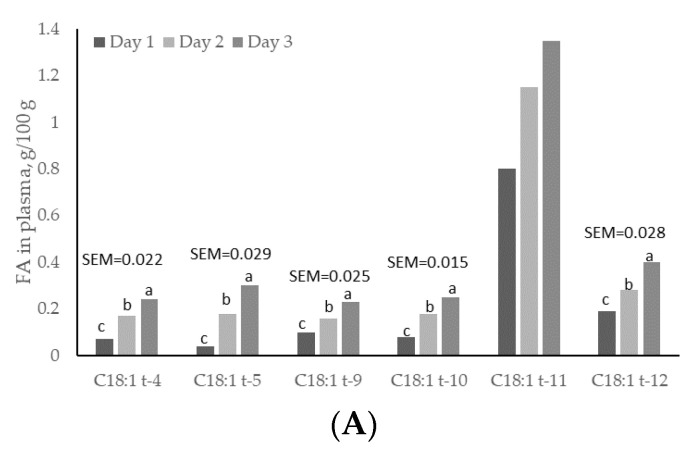

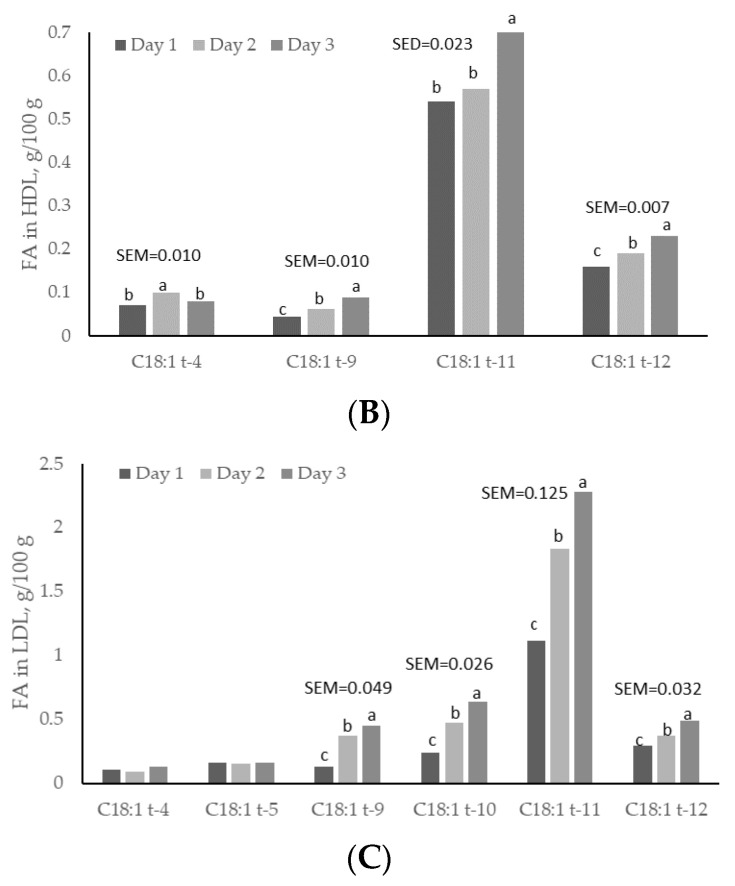
Day variations of C18:1 *trans* fatty acids (FAs) in (**A**) total plasma; (**B**) high-density lipoprotein (HDL) and (**C**) low-density lipoprotein (LDL) fractions from cows subjected to ruminal pulses of control, partially hydrogenated vegetable oil and soybean oil. SEM = standard error of the mean. ^a,b,c^ Means with different superscripts differ significantly (*p* < 0.05). Pooled means of control, partially hydrogenated vegetable oil and soybean oil per day are shown. *p*-value represents the effect of sampling day.

**Figure 3:**
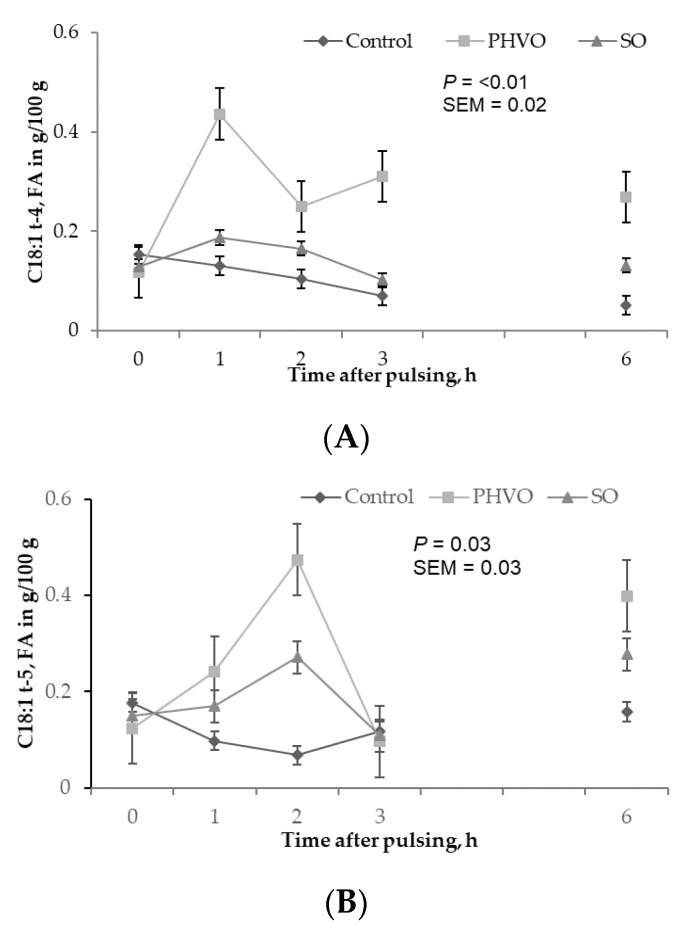

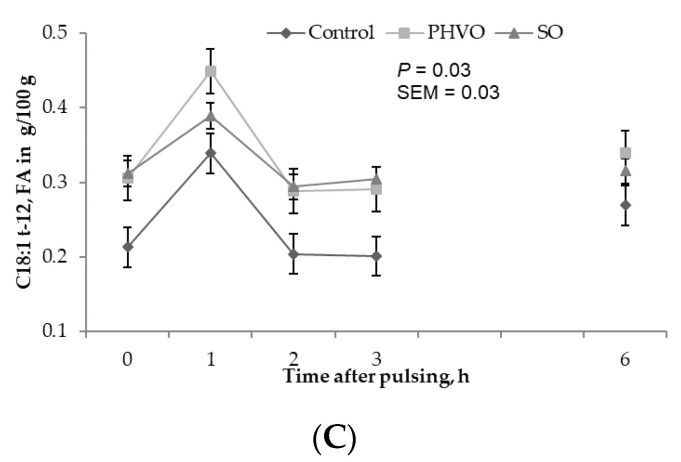
Time variations of C18:1 *trans* fatty acids in plasma from cows subjected to ruminal pulses of control, partially hydrogenated vegetable oil and soybean oil. (**A**) C18:1t-4; (**B**) C18:1t-5; and (**C**) C18:1t-12. Blood samples were collected prior to ruminal pulse (0 h; 09:00) and 1, 2, 3 and 6 h after ruminal pulse. SEM = standard error of the mean. Means are pooled across days for each cow. *p*-value represents the effect of sampling time.

Except for C18:1 *trans*-11, plasma concentrations of all C18:1 *trans* isomers increased linearly (*p* < 0.05) with day of treatment, and were higher (*p* < 0.05) on day 3 than on days 1 and 2 (Figure 2). C18:1 *trans*-11 was not increased in plasma but it showed a linear increased across sampling days. This *trans* isomer is crucial for formation of C18:2 *cis*-9, *trans*-11, and for dairy consumers this FA is important because it has potential benefits for human health [4]. Plasma concentrations of C18:1 *trans*-4 and C18:1 *trans*-12 were greater (*p* < 0.05) at 1 h post-rumen pulsing than at other times; concentrations of C18:1 *trans*-5 were greater (*p* < 0.05) at 2 h and 6 h post-rumen pulsing than at other times; concentrations of the remaining C18:1 *trans* isomers were not affected (*p* > 0.05) by time of sampling (Figure 3).

**Figure 4:**
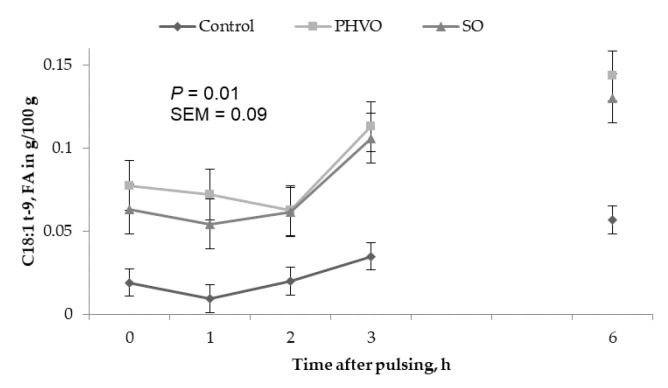
Time variations of C18:1t-9 in high density lipoprotein from cows subjected to ruminal pulses of control, partially hydrogenated vegetable oil and soybean oil. Blood samples were collected prior to ruminal pulse (0 h; 09:00) and 1, 2, 3 and 6 h after ruminal pulse. SEM = standard error of the mean. Means are pooled across days for each cow. *p*-value represents the effect of sampling time.

Concentrations of C18:1 *trans*-9, *trans*-11 and *trans*-12 in the HDL fraction increased linearly (*p* < 0.05) with day of treatment and were higher (*p* < 0.05) on day 3 than on days 1 and 2 (Figure 2). Concentrations of C18:1 *trans*-4 in the HDL fraction changed quadratically (*p* < 0.05); concentrations of C18:1 *trans*-9 were greater (*p* < 0.05) at 3 h and 6 h post-rumen pulsing than at other times; concentrations of the remaining C18:1 *trans* isomers were not affected (*p* > 0.05) by time of sampling (Figure 4).

There was a linear effect (*p* < 0.05) of the day on C18:1 *trans*-9, *trans*-10, *trans*-11 and *trans*-12 in the LDL fraction. These concentrations were higher (*p* < 0.05) on day 3 than on days 1 and 2, but concentrations of C18:1 *trans*-4 and *trans*-5 in the LDL fraction did not differ (*p* > 0.05) between days (Figure 2). Concentrations of C18:1 *trans* isomers in the LDL fraction were not affected (*p* > 0.05) by time of sampling. Sun and Gibbs [10] reported diurnal variation in concentration of C18:1 *trans*-11 in rumen digesta of grazing cows, with higher concentrations early in the morning from 02:00 to 06:00 h. In the current study, however, there was no time effect on concentration of C18:1 *trans*-11 in plasma and lipoprotein fractions. As rumen concentration and rumen production/uptake of C18:1 *trans*-11 are not always related, and rumen volume varies considerably in grass-fed cows, a consistent C18:1 *trans*-11 supply to the plasma is not precluded by having a rumen concentration flux across the day. C18:1 *trans*-11 can be converted to C18:2 *cis*-9, *trans*-11 (rumenic acid) through the action of stearoyl coenzyme A desaturase and it has been estimated that 20% of C18:1 *trans*-11 can be converted to C18:2 *cis*-9, *trans*-11 in humans [43]. Rumenic acid has been identified as anti-carcinogenic, anti-atherosclerotic, antioxidative and an immunomodulator [44]. The current study shows that C18:1 *trans*-11 did not vary across time after pulsing, but this isomer increased with days of treatment with both treatments. Thus, under the conditions of the current study, it may be advised to feed lactating cows with SO rather than PHVO, since PHVO can increase contents of other isomers such as C18:1 *trans*-10, which may reduce milk fat yield.

Although ruminal microbial activity was not measured in the current study, blood FA profiles were used as an indirect approach to visualize how infused FA were affected during rumen biohydrogenation [45]. Rumen pulses do not bypass biohydrogenation; hence, FA supply to the duodenum should be similar to supplies observed when oilseeds or oils are included in the diet. Rumen pulses are more representative of FA normally available to the animal than duodenal infusions. In the current study, however, a discrete lipid infusion (as opposed to lipids mixed with total mixed rations) may have provoked shifts in rumen motility and postprandial outflow, which may have influenced escape of rumen biohydrogenation products to the blood circulation. Post-ruminal infusions can be used to test hypotheses about intestinal uptake, but they only mimic rumen-protected lipids [46]. In on-farm conditions, there is a minimal loss of FA from the rumen by absorption either across the ruminal epithelium or by catabolism to VFA or CO_2_ [47]. In addition, microbes synthesize FA *de novo* from carbohydrate precursors. Therefore, lipids reaching the duodenum consist of FA from both dietary (around 87% coming from feed; [48]) and microbial origins [49].

The relatively short treatment (single administration per day), and sampling (3 d) periods in the current study were selected to provoke the extensive biohydrogenation effects on oil pulses that are often observed during the first few hours after administration [16]. The oil administration method can affect how long oil remains in the rumen. With a single administration of 250 g/d of oil in 500 mL/d of SM, plasma FA concentrations were higher during the first few hours after pulse and lower in subsequent hours. Generally, sampling day had a linear effect on C18:1 *trans* isomers in plasma, HDL and LDL. In the current study, effects of treatments on the TFA profile of plasma and lipoprotein fractions were observed within the first three hours post-pulse. Sampling day affected the TFA of plasma and lipoprotein fractions. Under on-farm conditions, ruminal metabolism of dietary lipids can be slower than with rumen pulses, and metabolism depends on feeding management (e.g., pasture-based diets vs. total mixed rations; varying forage to concentrate ratio) and production traits (e.g., body weight, milk yield and energy efficiency), among other variables.

The amount and composition of lipids circulating in the blood of dairy cattle are dependent upon a number of physiological variables; nature of the diet, time since feeding, age, breed, pregnancy and stage of lactation may all affect lipid content and composition [50]. In the current study, diurnal variations of plasma, lipoprotein fractions and ruminal fermentation parameters suggested there might be adaptation of ruminal microbiome to treatments [51]. Future studies should focus on the rumen microbiome and consider longer treatment periods to test this hypothesis.

Identification of key C18:1 *trans* isomers during the daily feeding cycle could be used to evaluate production of intermediates from different polyunsaturated oils with less risk of bias than in vitro trials [11]. In on-farm conditions, cows may be fed with different sources of polyunsaturated FA, such as oilseed by-products, that behave similarly to observations in the current study. Furthermore, cows might be fed with bypass fats that are usually made from hydrogenated vegetable oils [52]. Under the conditions of the current study, concentrations of C18:1 *trans* isomers appeared to be higher in the first 2 h post-rumen pulsing, then decreased after 2 h post-pulsing and finally increased steadily. The day and time responses observed in the current study were linked to the level of supplemented oil, the degree of accumulated unesterified FA, and rumen microbial adaptations [53]. In order to have a clear picture of the metabolic pathways for C18:1 *trans* isomers in the rumen, plasma, HDL and/or LDL, future work will need to consider long-term studies, lactating cows, rumen microbiome data, and milk FA profiles. Milk fats consist mainly of triglycerides (TAG; 98%), and other types of lipids such as diacylglycerides (2% of the lipid fraction), cholesterol (less than 0.5%), polar lipids (1%, mainly phospholipids) and free FA (0.1%) [5]. Given that the mammary gland preferentially takes up FA from plasma TAG, it is recommended for future research to study FA accumulation of C18:1 TFA either as total plasma TAG or TAG concentrations in each lipoprotein fraction.

### 3.5. Further Considerations

The current study was designed to study short-term variations in plasma, lipoprotein fractions and ruminal fermentation parameters, which would be needed for mammary absorption studies in lactating cows. This study sheds light on the early-stage kinetics of plasma FA transport and rumen fermentation kinetics, which contributes to our understanding of long-term nutrition in dry cows that need to adapt to different metabolic challenges in the following early lactation phase. In this regard, our results showed that dietary lipids undergo extensive changes during the first hour post-pulse and in plasma and lipoproteins the majority of C18:1 TFA increased towards the third day after consecutive rumen pulses. Direct extrapolation of these results to lactating cows fed on total mixed rations is difficult as physiological state and energetic balance will differ, as will rates of FA influx and outflow from the rumen. However, differential effects of lipid sources on plasma FA were generally seen within one hour of pulsing, and most differences were maintained for 3 h. This suggests that administration of different lipids in total mixed rations could lead to similar effects, but at lower magnitudes and more prolonged. Further research is required to test this suggestion.

Some additional factors need to be considered for interpretation of the present data. (1) Due to our methodological approach, it was not possible to separate the effect of time post-feeding and the effect of time post-administration of lipids (instead, control treatment could be used as a measure for this effect, or at least to identify effects of basal diet fermentation vs. oils). (2) The animals had *ad libitum* access to hay, but the commercial concentrate was offered at 12:00 (3 h after the pulse of lipids). Thus, the slight variations in fermentation parameters between 0 and 3 h (up to 3 h post-rumen pulsing) may be explained by hay intake, and the greater decrease in ruminal pH at 6 h post-rumen pulsing after the pulse of lipids would probably be attributable to concentrate intake. (3) Effects of sampling time on plasma fatty acid profiles may also be influenced by the feeding schedule.

## 4. Conclusions

In summary, the results showed that there was an accumulation of several C18:1 TFA in plasma and lipoproteins, especially on the third day of rumen pulsing. Each C18:1 TFA responded differently to treatments over time. Our data show that naturally occurring C18:1 TFA isomers (produced during rumen biohydrogenation of SO) and preformed TFA (supplied by PHVO) elicit differential FA partitioning and transport in plasma and lipoproteins.

## Figures and Tables

**Table 1:** Ingredients and declared chemical composition of the concentrate.

	Concentrate ^1^
Ingredient composition (% of dry matter)	
Rolled barley	50
Sugar beet pulp	15
Rolled wheat	15
Cane molasses	5
Limestone flour	5
Vitamins	5
Minerals	5
Chemical composition (% of dry matter)	
Neutral detergent fiber	8
Crude protein	14
Ether extract	3
Ash	7

^1^ Commercial concentrate from Manor Farm Feeds, Oakham, UK.

**Table 2:** Fatty acid composition of oils, skimmed milk and emulsions used for ruminal pulses.

Oils			Treatment Emulsions ^4^		
Fatty Acid, g/100 g	SO ^1^	PHVO ^2^	Control ^3^	SO + SM	PHVO + SM
C10:0	-	-	2.51	-	-
C12:0	-	0.23	2.14	-	0.24
C14:0	0.07	0.18	7.89	0.08	0.30
C14:1	-	-	0.50	-	-
C15:0	-	-	0.98	-	-
C16:0	10.74	7.82	27.02	10.84	7.80
C16:1	-	-	1.54	-	0.13
C17:0	-	-	0.63	0.08	0.07
C18:0	4.28	8.35	11.69	4.30	8.15
C18:1 *trans*-4	-	0.59	-	-	0.48
C18:1 *trans*-5	-	1.48	-	-	1.38
C18:1 *trans*-6–8	-	8.39	-	-	8.33
C18:1 *trans*-9	-	17.60	-	-	16.67
C18:1 *trans*-10	-	9.28	-	-	8.96
C18:1 *trans*-11	-	7.67	1.70	-	7.54
C18:1 *trans*-12	-	7.35	-	-	6.97
C18:1 *cis*-9	22.42	6.06	21.08	22.33	6.27
C18:2 *trans*-9, cis-12	-	1.13	-	-	0.84
C18:2 *cis*-9, *cis*-12	53.03	0.42	3.39	52.90	0.46
C20:0	0.34	0.76	-	0.34	0.73
C18:3 *cis*-6, 9, 12	-	0.42	-	-	0.58
C20:1	0.20	-	-	0.18	0.15
C18:3 *cis*-9, 12, 15	6.78	0.06	0.61	6.75	0.14
C18:2 *cis*-9, *trans*-11	-	-	1.03	-	-
C20:2	0.05	0.05	-	-	-
C22:0	0.38	0.44	-	0.37	0.42
C24:0	0.15	0.15	-	0.14	0.18
Other ^5^	1.56	21.57	17.29	1.69	23.21

^1^ SO = soybean oil; ^2^ PHVO = partially hydrogenated vegetable oil; ^3^ SM = skim milk; ^4^ single administration per day, ^5^ fatty acids < 0.03 g/100 g.

## Data Availability

The data presented in this study are available on request from the corresponding author.
